# Application of a Single Cell Electric-SRR Metamaterial for Strain Evaluation

**DOI:** 10.3390/ma15010291

**Published:** 2021-12-31

**Authors:** Michal Herbko, Przemyslaw Lopato

**Affiliations:** Center for Electromagnetic Fields Engineering and High-Frequency Techniques, Faculty of Electrical Engineering, West Pomeranian University of Technology, ul. Sikorskiego 37, 70-313 Szczecin, Poland

**Keywords:** metamaterial, metasurface, split ring resonator (SRR), single cell, strain sensor, structural health monitoring (SHM)

## Abstract

Strain is a crucial assessment parameter in structural health monitoring systems. Microstrip sensors have been one of the new types of sensors used to measure this parameter in recent years. So far, the strain directionality of these sensors and the methods of miniaturization have been studied. This article proposes the use of a single cell metamaterial as a resonator of the microstrip sensor excited through the microstrip line. The proposed solution allowed for significant miniaturization of the microstrip sensor, with just a slight decrease in sensitivity. The proposed sensor can be used to measure local deformation values and in places with a small access area. The presented sensor was validated using numerical and experimental methods. In addition, it was compared with a reference (rectangular geometry) microstrip sensor.

## 1. Introduction

The safety and integrity of infrastructure is an important aspect of building and maintaining activities. Therefore, Structural Health Monitoring (SHM) systems are becoming more and more popular. SHM systems support or even replace periodic structural condition inspections. The implementation of SHM in various structures is beneficial in many ways such as increasing public safety, cutting costs of exploitation and monitoring, improving the life span of constructions, and early detection of risks. This leads to improvement of the examined object’s overall performance [1]. Large dimensions of structures, fancy shapes, new building materials, as well as monitoring of old structures have resulted in increased expenses on SHM systems and their development. Extensive research is being carried out to improve efficiency and reduce the cost of SHM systems. In particular, research is conducted on the topics of new sensors, communication of system components, and data processing including exploitation time estimation.

Strain sensors are a crucial component in most sensor networks used to monitor civil structures. In the case of bridge condition monitoring systems, about 50% of all sensors are strain sensing elements [2]. The stress measurement of steel structures is performed in two ways. The first method is the analysis of the magnetic properties of steel using a magnetic sensor [3]. The second option is to use a sensor attached to the tested structure. The sensor output signal depends on the deformation. Until now, stress sensors have been invented using various physical phenomena. The first strain gauge was the proposed and commercialized resistance strain gauge by Edward E. Simons and Arthur C. Ruge [4]. The operating principle of a resistance strain gauge is based on the changes in a conductor’s electrical resistance as a result of changes in its cross-sectional area and length. The resistance value of the sensor increases as it is stretched. The change in the resistance of a semiconductor element is used in piezoresistive sensors. In this case, the crystal lattice of the semiconductor is distorted, which affects the energy bands [5]. Utilization of the piezoelectric effect in the strain sensor is presented in [6]. In the case of FBG (Fiber Bragg Grating) sensors, deformation changes the length of the reflected light patch [7].

Microstrip antennas for strain measurements have been proposed and developed in recent years [8,9,10,11,12,13,14,15,16,17,18,19,20,21,22,23,24,25]. The vector network analyzer (VNA) measures the frequency domain reflection coefficient (*S*_11_) of the microstrip antenna sensor. The entire measurement system should be considered as a uniform transmission line. All components of the system (VNA port, coaxial wires, SMA connector, microstrip line) have the same impedance (usually equal to 50 Ohms). When a transmission line is loaded with wave impedance, the signal given at the input is not reflected. Planar microwave resonators (patch, radiating part of antenna sensor) have an impedance equal to the wave impedance at resonant frequencies. The resonant frequency depends on the shape and size of the patch. Changing the shape of the patch (i.e., because of external strain) changes the resonant frequency. In this type of sensor, the reflection coefficient as a function of frequency is monitored to determine the deformation based on the resonant frequency shifts. The sensors were fed in wired form [8,9,10,11,12,13,14,16,17,18,19,20,22,24,25] and wireless [15,21,23]. The first and most studied sensor geometry is the rectangular microstrip strain sensor [8,9,10,11,12,18,19,20,21,24]. This geometry provides the highest sensitivity at a given resonance frequency. In the work of [24], rectangular microstrip sensors were used to measure deformations. The patches were designed for different resonant frequencies. The following conclusions can be drawn from the study: the higher the resonant frequency, the bigger its shift, and the smaller the size of the sensor. Additionally, circular [13,14,15,25] and other shapes [16,17,22,23,26] of patches were utilized. Other sensor geometries have been developed for the application of local strain measurements or for measuring the direction and value of strain. An important advantage of microstrip sensors is the ability to measure many resonance frequencies with different current density distributions in the patch. This property allows the direction and stress values to be measured using one sensor. For other types of strain gauges, several sensors must be used to measure the direction and strain value. For this purpose, sensors with a rectangular [20] and circular patch [13] and two rectangular patches powered by a T-junction power divider [22] were used.

The patches dimensions range from 0.5λ to 6λ (λ—wavelength) depending on the utilized geometry. Selected applications require a smaller sensor. This necessity results from measurements of local deformation values or in places where there is a small measurement area. Microstrip strain sensors designed for higher operating frequencies are smaller and have higher sensitivity. So it seems that the best way is to design sensors for high resonant frequencies. Notwithstanding, the prices of Vector Network Analyzers strongly depend on their maximum frequency, which makes the use of sensors with very high operating frequencies economically unjustified. Microstrip deformation sensors were miniaturized using the following methods:

- application of a laminate with high electric permittivity [25],
- utilization of a special patch geometry [17].

The work of [25] investigated the sensitivity of circular microstrip strain gauges designed on various laminates. Similar resonant frequency shifts were obtained for all sensors. The use of a high electric permittivity laminate (*ε*_r_ = 13.2) made it possible to significantly reduce the size of the sensor. The patch radius length was 9.68 mm, while for *ε*_r_ = 2.2, the radius length equaled 23.7 mm. Application of the specific patch geometry can also reduce the sensor dimensions. This miniaturization method was carried out by applying the Sierpinski curve fractal in [17]. The sensitivity of the sensor for three iterations of this fractal was tested. The higher the fractal iteration, the smaller the size and sensitivity. For the third iteration of this fractal, the patch size was reduced four times, with a two-fold reduction in sensitivity in relation to the rectangular resonator.

This article presents further research on the miniaturization of microstrip sensors by selecting the appropriate patch geometry. A single metamaterial element was used in this work. Metamaterials are man-made artificial structures that enable material properties to be obtained that do not occur in nature, e.g., negative refractive index n, consequently negative electric permittivity *ε*_r_ and magnetic permittivity *µ*_r_ [26,27,28,29]. They consist of a 2- or 3-dimensional matrix of structural elements (cells) with dimensions several times smaller than the electromagnetic wavelength at which they are supposed to work. Their unique resonance properties can be relatively easily controlled by appropriately designing the geometry of the structural elements. In this work, one of the best-known metamaterial structures—a variant of the split ring resonator (SRR)—was used. In the literature, this variant is called a double split-ring resonator (dSRR). In this work, the proposed sensor was compared with a rectangular microstrip sensor. Numerical calculations and strain measurements were performed in order to evaluate the designed sensors.

## 2. Sensors Design

In this work, the topic of miniaturization of microstrip sensors has been developed. This is especially important when measuring local strain values or where there is not enough places to attach the sensor. We can reduce the dimensions of the patch by using a patch operating at a higher operating frequency. However, this solution has disadvantageous effects because it is necessary to use a Vector Network Analyzer with a higher measuring frequency range. One of the methods of reducing the size of the sensor is the use of a laminate with high electric permittivity [25]. Another solution is to use the appropriate shape of the patch. The work of [17] uses a patch in the shape of a Sierpinski curve. In this work, it was decided to use a single structural element of the metamaterial as the patch of the microstrip strain sensor. For this purpose, a double Split-Ring Resonator (dSRR) was chosen. This choice resulted from the possibility of easy matching of the patch impedance to the microstrip line using the inset feed. This choice of the impedance matching method resulted from the possibility of limiting the measuring area and the size of the sensor. Narrow-band impedance matching can be also achieved by a quarter wave transformer and stubs. But these solutions increase the size of the sensor and have the potential to affect the accuracy of local strain value measurements. The sensor is designed using an optimization method for a resonant frequency *f*_r_ = 2.725 GHz.

**Figure 1:**
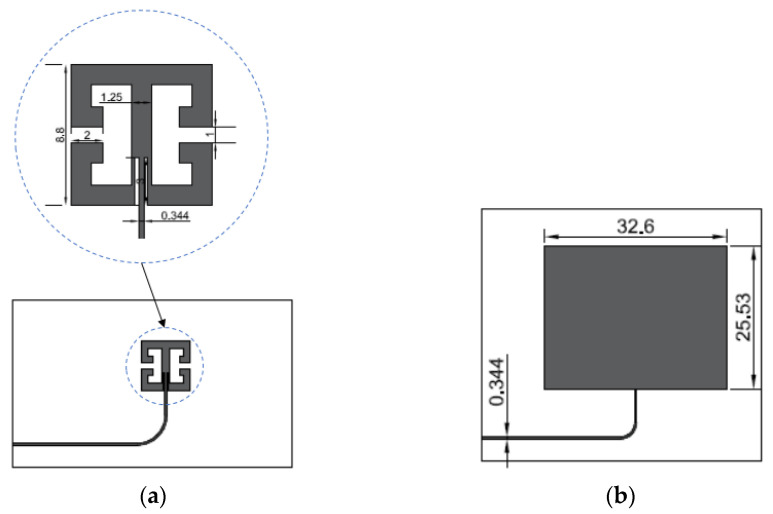
Sensor design (**a**) Double split-ring resonator (dSRR) microstrip sensor; (**b**) Rectangular microstrip sensor (dimensions in mm).

A rectangular microstrip sensor was designed for the same operating frequency [30]. This assumption avoids the influence of the resonant frequency on the sensitivity of the microstrip transducer. All sensors were designed on glass-reinforced epoxy laminate FR4 (main dielectric parameters: *ε*_r_ = 4.4, tanδ = 0.02). The laminate was 0.18 mm thick. The dimensions of the sensors are shown in Figure 1.

The transducer is glued to the element under test (planar sample). As a result of the action of external mechanical forces on the sample, the deformation is transferred through the adhesive connection to the evaluated sensor. The flexibility of utilized FR4 laminate is enough for measured strains—in performed experiments within previous works, the evaluation was done even for plastic deformations of samples (which is far too much in the case of standard SHM applications). The resonant frequency shift is due to deformation-induced changes in the geometry of the resonator. It depends on the direction of deformation: geometric dimensions parallel to the axis of the acting force increase, while perpendicular dimensions decrease. This causes a change in the distribution of electromagnetic fields and the flow of currents, which is reflected in slight changes in the equivalent circuit of such a system as well, as in the frequency response of the sensor’s reflection coefficient and consequently the resonant frequency. Current loops dimension changes influence the inductance of the element, while electric field gap (between conductors) changes influence the capacitance of the resonator element. Thus, the principle of operation does not differ in any way from the principle of operation of commonly used microstrip resonators.

## 3. Sensors Validation

Numerical analysis and experimental validation were conducted for the designed sensors. The description will be presented in this section.

### 3.1. Numerical Analysis

**Figure 2:**
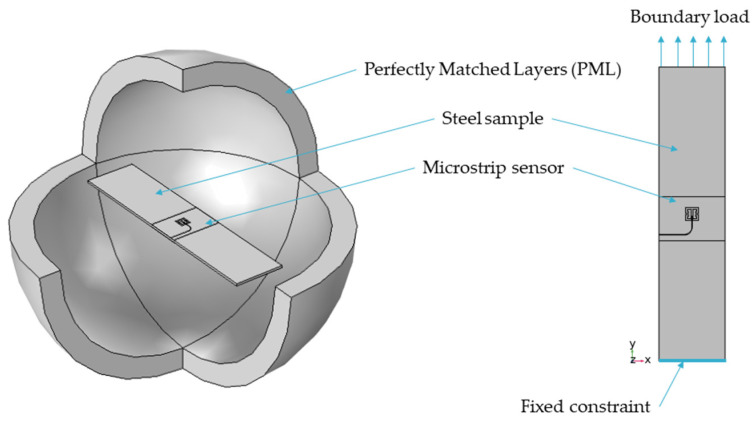
Finite Element Method model with a dSRR sensor attached to the steel sample.

The Finite Element Method (FEM) model was developed in the Comsol Multiphysics environment to evaluate the designed sensors. The numerical model geometry is shown in Figure 2. The designed microstrip sensor was fixed to the S355J2+N construction steel planar sample. This material is often utilized in civil constructions. The dimensions of the samples utilized were length 250 mm, width 45 mm, and thickness 1 mm. At first, the Solid Mechanics module was used to compute the deformation of the steel sample. In this case, the following equation was solved using FEM [31]:

(1) $$0=\nabla \cdot S+F_{V}$$

where: *S*—second Piola-Kirchhoff stress tensor, **F**_v_—force per unit volume.

Mechanical loading of the sample simultaneously deforms the tested microstrip sensor. It causes the change of the current density distribution in the microstrip patch as well as the *S*_11_ frequency domain response. The electromagnetic problem was computed using the RF module, where the following equation was solved [32]:

(2) $$\nabla \times \mu_{r}^{-1}\left(\nabla \times E\right)-k_{0}^{2}\left(\varepsilon_{r}-\frac{j\sigma_{e}}{\omega \varepsilon_{0}}\right)E=0$$

where: *σ*—electric conductivity, *ε*_r_—relative permittivity, *µ*_r_—relative permeability, *k*_0_—wave number, **E**—electric field, *ω*—angular frequency.

**Figure 3:**
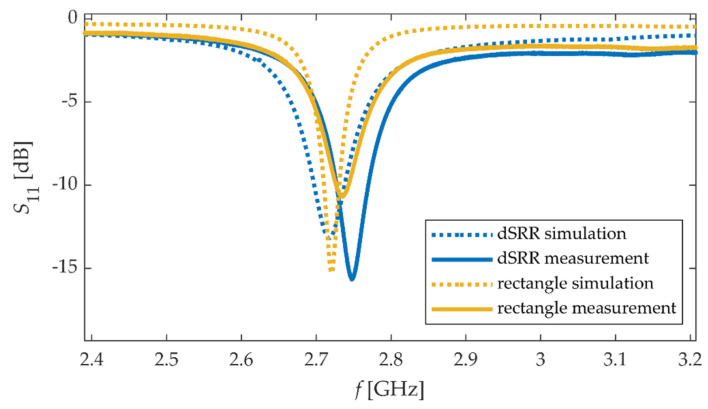
Calculated and measured module of reflection coefficient *S*_11_ for considered sensors.

The sensors’ resonant frequency was determined based on the estimation of the local minimum of the obtained magnitude of the reflection coefficient *S*_11_ characteristics. Received *S*_11_ frequency responses obtained using calculations and measurements are shown in Figure 3.

### 3.2. Experimental Validation

**Figure 4:**
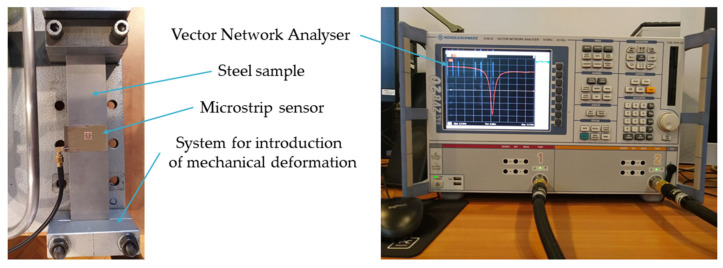
Photo of the measuring system.

In this section, experimental evaluation of designed and simulated sensors was conducted. In this case, dSRR and rectangular sensors were manufactured using the photolithographic process. Dimensions of designed sensors are shown in Figure 1. Tested sensors were fixed with cyanoacrylate adhesive to the construction steel samples. This adhesive connection allowed the transmission of sample strain to sensors. The sample was extended by a custom mechanical deformation system. Next, Rohde & Schwarz ZVB20 Vector Network Analyzer (Columbia, MD, USA) was used for *S*_11_ acquisition (in the case of the deformed sensor). Measurements were carried out in the 2.3–3.3 GHz frequency range with 0.1 MHz steps. The utilized power level was 0 dBm. The photo of the measurement setup used for this purpose is shown in Figure 4. Until recently, Vector Network Analyzers were devices with large dimensions and high prices. In recent years, small and low-cost VNAs have been developed, which could be used in the future in practical SHM measurements. In our research, we used a frequency range (<3 GHz) that allowed the use of such low-cost VNAs.

### 3.3. Results

The deformation assessment is performed by monitoring the frequency response of the reflection coefficient *S*_11_ magnitude. In order to determine the deformation, it is necessary to determine the shift of the resonance frequency Δ*f*_r_ from the initial state.

(3) $$\Delta f_{r}=f_{\mathrm{rload}}-f_{r\varepsilon =0}$$

where: *f*_rload_ is the resonant frequency for the case with external mechanical load, and *f*_rε=0_ is the resonant frequency without mechanical deformation.

**Figure 5:**
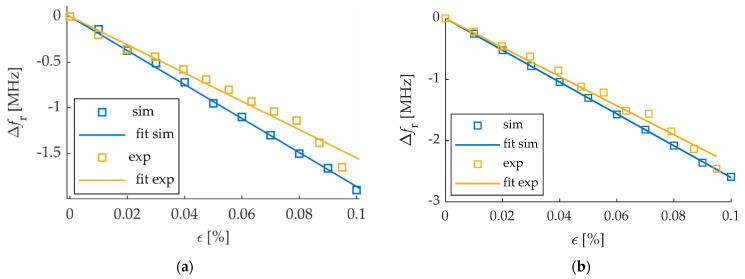
Influence of strain *ε* on shifts of resonant frequency Δ*f*_r_ (**a**) dSRR microstrip sensor; (**b**) Rectangular microstrip sensor.

**Figure 6:**
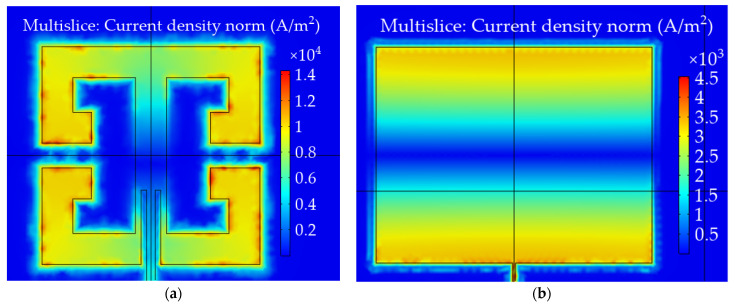
Current density [A/m^2^] distribution in case of resonant frequency (**a**) dSRR microstrip sensor; (**b**) rectangular microstrip sensor.

**Figure 7:**
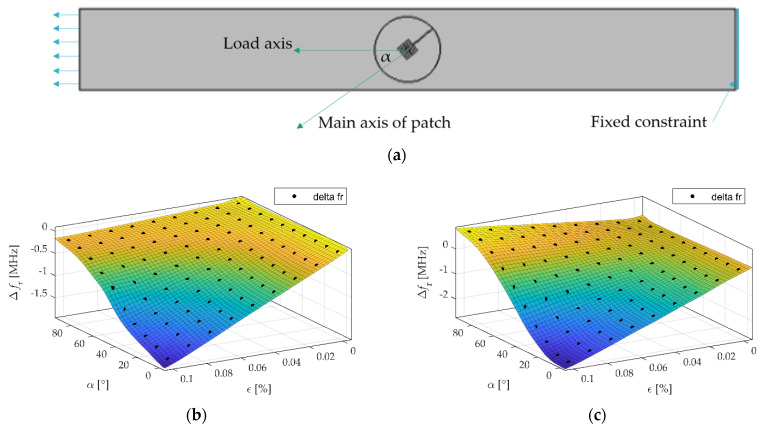
Directional characteristics of Δ*f*_r_ for different strain levels (**a**) top view of the considered sample and definition of applied strain direction angle *α*; (**b**) dSRR microstrip sensor; (**c**) rectangular microstrip sensor.

The resonant frequency is the frequency at which the local minimum of the magnitude of *S*_11_ occurs. In order to determine the resonance frequency of the transducer, in the first step, the frequency range with the local minimum of the reflection coefficient magnitude was selected. Then a two-term Gaussian model was used to fit the measured data to the mathematical equation, and based on that, the resonant frequency was designated. The resonant frequency shifts were determined using the numerical method and measurement for the dSRR microstrip sensor and the rectangular patch. The relationships between material strain and the obtained resonant frequency shifts for the strain along the main patch axis are shown in Figure 5. Comparing the results shown in Figure 3 and Figure 5, a good convergence between simulations results and measurement was obtained. The sensitivity of the dSRR patch is lower than the rectangular microstrip sensor by 28% in simulations and 35% in measurement. The utilization of the dSRR patch made it possible to reduce the patch size by 93% (Table 1). The lower sensitivity of the dSRR sensor results from the current density distribution at a studied resonance. In the case of the rectangular patch, the current direction is parallel to the current density distribution at the first resonance. In the case of the dSRR patch, the current distribution is more multidirectional as shown in Figure 6. Moreover, the strain directional characteristics of the tested sensors were determined, as shown in Figure 7. The force direction strongly influences the change of the studied resonance frequencies. Therefore, a single resonance measurement of the deformation with these sensors can only be performed for a known strain direction.

In the case of rectangular sensor and direction angle *α* = 0°, increasing the strain causes a rapid decrease of Δ*f*_r_, but for the direction angle *α* = 90°, the Δ*f*_r_ is slightly increasing. This is caused by an elongation of the sample in the direction parallel to the load, and its shortening in the perpendicular direction. This has an important implication: there is some direction, for which there will be no change of Δ*f*_r_ caused by strain, thus the sensor will be not sensitive to the strain. This restricts the application of the rectangular sensor to applications where the direction of external load is unknown. In the case of the proposed sensor, for both directions (*α* = 0° and *α* = 90°), an increase of strain causes a decrease of Δ*f*_r_. Thus, there will be no such case where the proposed sensor is not sensitive to any strain.

## 4. Conclusions

In this article, the dSRR microstrip strain sensor has been designed and tested. The proposed sensor has been compared with a rectangular microstrip sensor. Both are designed for the same resonant frequency. This assumption eliminates the influence of the resonant frequency on the sensitivity of the transducer. So only the patch shape effect is investigated. The sensors were verified by numerical and experimental analysis. The proposed geometry allowed for the reduction of the patch size by over 90% with a simultaneous approx. There was a 30% decrease in sensitivity in relation to the rectangular resonator. This is a better result than that obtained in [17], where a 75% reduction in the patch size was achieved with a two-fold decrease in sensitivity in relation to the rectangular resonator. Table 2 shows a comparison of the sensitivity of different microstrip transducers. As can be seen, the resonant frequency, as well as the shape of the resonator, affect the sensitivity of the sensor. For similar operating frequencies (2.4–3.0 GHz—the band covered by low-cost VNAs) the dSRR sensor proposed in this paper has the best sensitivity to patch size ratio.

However, it is still a worse method of miniaturization of microstrip stress sensors than the utilization of laminate with high electric permittivity. The proposed method of miniaturization can be used in conjunction with a laminate with high electric permittivity, in applications where the sensitivity requirements are lower and where deformation tests on a small area are required. Moreover, the proposed sensor is devoid of one of the drawbacks of the rectangular sensor—the sensitivity does not drop to zero at the selected load direction. The obtained results are promising and allow us to state that strain sensors built on single structural elements of metamaterials or their small collections have great potential. Further work will consist of further miniaturization of the transducers and increasing their sensitivity. In the future, we also plan to focus on wireless strain sensors or sensor pairs. For this reason, the research will be extended to include the transmission coefficient and group delay. Additionally, an analysis based on 3D Smith charts [33,34] is planned.

## Figures and Tables

**Table 1:** Comparison of the studied sensors.

Patch Shape	Sensitivity [kHz/µε]—Simulation	Sensitivity [kHz/µε]—Measurement	Resonator Size [mm^2^]
dSRR	−1.862	−1.548	77.44
Rectangle	−2.602	−2.379	832.28

**Table 2:** Comparison of recently reported microstrip strain sensors.

Reference	Patch Shape	Frequency [GHz]	Sensitivity [kHz/µε]	Patch Size [mm^2^]	Sensitivity/ Patch Size Ratio [kHz/µε·mm^2^]	Dielectric
This work	rectangular	2.725	−2.379	832.28	−0.002858	FR4
This work	dSRR	2.725	−1.548	77.44	−0.019990	FR4
[8]	rectangular	17.2	−17.2	21.32	−0.806754	Kapton
[10]	rectangular	3	−2.54	N/A	N/A	RT Duroid 5880
[13]	circular	2.5	−2.05	1123.59	−0.001825	FR4
[17]	first iteration of Sierpinski curve fractal	2.725	−2.35	778.24	−0.003020	FR4
[17]	second iteration of Sierpinski curve fractal	2.725	−1.36	333.54	−0.004077	FR4
[17]	third iteration of Sierpinski curve fractal	2.725	−1.18	184.59	−0.006393	FR4
[18]	rectangular	2.469	−2.847	1138.36	−0.002501	FR4
[19]	rectangular	7.31	−3.43	750	−0.004573	PDMS
[22]	double patch sensor	2.75	−2.82	823.28	−0.003425	FR4

## Data Availability

Not applicable.
